# Minimally invasive, patient specific, beat-by-beat estimation of left ventricular time varying elastance

**DOI:** 10.1186/s12938-017-0338-7

**Published:** 2017-04-13

**Authors:** Shaun Davidson, Chris Pretty, Antoine Pironet, Shun Kamoi, Joel Balmer, Thomas Desaive, J. Geoffrey Chase

**Affiliations:** 1 Department of Mechanical Engineering, University of Canterbury, Christchurch, New Zealand; 2 GIGA-Cardiovascular Sciences, University of Liège, Liège, Belgium

**Keywords:** Time varying elastance, Cardiovascular system, Minimally invasive

## Abstract

**Background:**

The aim of this paper was to establish a minimally invasive method for deriving the left ventricular time varying elastance (TVE) curve beat-by-beat, the monitoring of which’s inter-beat evolution could add significant new data and insight to improve diagnosis and treatment. The method developed uses the clinically available inputs of aortic pressure, heart rate and baseline end-systolic volume (via echocardiography) to determine the outputs of left ventricular pressure, volume and dead space volume, and thus the TVE curve. This approach avoids directly assuming the shape of the TVE curve, allowing more effective capture of intra- and inter-patient variability.

**Results:**

The resulting TVE curve was experimentally validated against the TVE curve as derived from experimentally measured left ventricular pressure and volume in animal models, a data set encompassing 46,318 heartbeats across 5 Piétrain pigs. This simulated TVE curve was able to effectively approximate the measured TVE curve, with an overall median absolute error of 11.4% and overall median signed error of −2.5%.

**Conclusions:**

The use of clinically available inputs means there is potential for real-time implementation of the method at the patient bedside. Thus the method could be used to provide additional, patient specific information on intra- and inter-beat variation in heart function.

**Electronic supplementary material:**

The online version of this article (doi:10.1186/s12938-017-0338-7) contains supplementary material, which is available to authorized users.

## Background

Cardiovascular disease and dysfunction (CVD) are leading causes of Intensive Care Unit (ICU) admission and mortality worldwide. CVD was responsible for 31% of global deaths in 2013, and the estimated worldwide cost associated with CVD in 2010 was $863 billion USD, accounting for approximately 1.39% of gross world product in the same year [1]. Despite the need with aging populations for optimised cardiovascular care in the ICU, inadequate or incorrect diagnosis of cardiac disturbances resulting in increased length of stay, cost and mortality is an ongoing issue [2, 3]. Cardiac management in the ICU is often informed by measurements taken from catheters placed near the heart. However, despite the rich information available from such catheter waveforms, their use is not necessarily associated with improved clinical outcomes [4, 5]. Hence, improvement in the extraction of cardiac information from these catheter waveforms has the potential to yield value from readily available data that has potentially been under-utilised to date.

Time varying elastance (TVE) is an important means of expressing internal cardiac dynamics and function [6]. The TVE curve represents the active elastance changes in the ventricles that drive heart contraction, thus providing valuable intra-beat information about cardiac behaviour and energetics [6–8]. The TVE curve has a wide range of potential applications. It is often employed as an input in large cardiovascular models that seek to model the entire circulatory system [9–13], and also frequently used in the determining of important cardiac metrics, such as the end-systolic pressure–volume relation (ESPVR) [7].

There has been more limited investigation into the utility of the TVE curve in isolation. The area under the curve of the TVE curve is analogous to work done by the ventricle. It is thus a potentially significant indicator of patient condition. However, the typical normalisation of the TVE curve means only relative changes in work for a given inotropic state can be compared. The TVE curve relies on a combination of ventricular pressure and volume waveforms similar to pressure–volume (P–V) loops. It thus contains information concerning cardiac work [14, 15] and contractility [6, 16], both of which change in response to cardiac dysfunction. As such, it has been suggested that the shape of the ventricular elastance curve itself may have diagnostic use [17–19].

Unfortunately, the TVE curve can only be directly measured by placing catheters into the heart chambers, which is, understandably, not common practice. As such, the traditional approach to non-invasively generate a TVE curve is to fix it to a population based waveform [7, 11, 12]. Most existing work surrounding TVE curves is focused on using the TVE curve to estimate a clinical parameter, such as ejection fraction [20], and, most commonly, end-systolic elastance [7, 21, 22]. However, these studies are validated on correlations with the derived parameter, rather than on the shape and change in shape of the TVE curve itself. Thus, these approaches are not validated for use as part of a larger model of cardiac dynamics, or for direct use as a diagnostic aid.

Previous work specifically focused on experimentally generating a TVE curve and validating it based on its correlation with the analytically derived function showed promise, and noted changes in the TVE curves during pulmonary embolism and septic shock [17]. However, this work was limited by the availability of data for validation and the reliance on an assumed driver shape [17, 18]. Work has also been undertaken in modelling time varying ventricular elastance, split into active and passive components, in humans [19]. Active elastance was shown to compare well with other metrics of contractility, and the properties of these elastance curves were shown to change for different disease states. However, this method required highly invasive ventricular catheterisation and thus is not broadly implementable in a clinical environment. Hence, there is a significant gap created by the current clinical inability to directly measure or estimate the TVE curve every beat.

This paper presents a novel, minimally invasive method for deriving the TVE curve beat-by-beat. The method focuses on combining simple physiological assumptions with clinically available catheter waveforms to individually simulate the pressure and volume components that define the TVE curve, rather than generating the TVE curve itself. Importantly, this approach avoids the need to directly assume a shape for the TVE curve, and is thus better equipped to capture variations in this shape over time and condition, as well as the corresponding alterations in intra-beat cardiac behaviour.

Clinically feasible measurements mean the method has the potential for real-time implementation at the patient bedside, without requiring additional, invasive instrumentation. Such a TVE curve could be used to provide additional, patient specific information on intra-beat behaviour and inter-beat variation in the functioning of the heart. Finally, monitoring the driver’s evolution over time could add significant new data and insight to improve diagnosis and treatment.

## Methods

### Proposed method

The TVE curve is defined:

1 $$e(t) = \frac{{P_{lv} \left( t \right)}}{{V_{lv} \left( t \right) - V_{d} }}$$

where *P*_*lv*_ is the pressure in the left ventricle, *V*_*lv*_ is the volume in the left ventricle and *V*_*d*_ is the ‘dead space’ volume in the ventricle [6, 23]. Thus, the TVE curve is defined by two waveforms (*P*_*lv*_ and *V*_*lv*_) and one constant (*V*_*d*_). These values can be measured directly, but doing so is not clinically feasible [24].

**Fig. 1:**
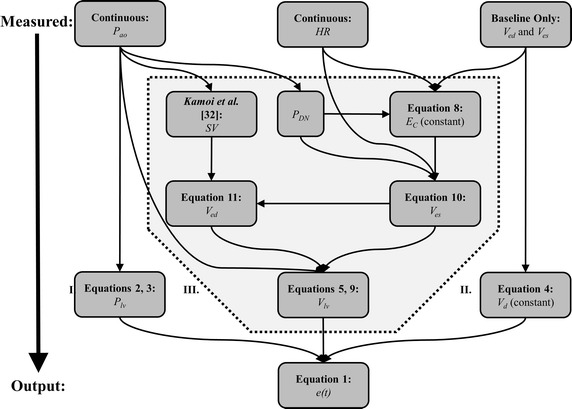
Flowchart of the proposed method. *Roman numerals* indicate different method regions

The proposed method approximates these waveforms (*V*_*lv*_, *P*_*lv*_) and constant (*V*_*d*_) using three inputs, as shown in Fig. 1. These inputs include continuously sampled aortic pressure waveforms (*P*_*ao*_) and heart rate (*HR*), data which is typically available in a modern ICU. The final input required is baseline end-systolic (*V*_*es*_) and end-diastolic (*V*_*ed*_) Volume, obtained from a brief echocardiography reading, which is increasingly available in a clinical setting [25].

The overall goal of this method is to use the clinically available inputs *P*_*ao*_, *HR* and baseline *V*_*es*_ and *V*_*ed*_ to determine the outputs *P*_*lv*_, *V*_*d*_ and *V*_*lv*_, and thus the TVE curve *e*(*t*) as set out in Fig. 1 and Eq. 1. This resulting TVE curve can be experimentally validated against the TVE curves derived from experimentally measured *P*_*lv*_ and *V*_*lv*_ in animal models. The availability of a nearby continuous pressure measurement (*P*_*ao*_) forms an effective basis for the continuous approximation of *P*_*lv*_, and the availability of a baseline volume measurement (*V*_*es*_) forms an effective basis for the approximation of baseline *V*_*d*_. However, there is no continuous volume measurement available for continuous approximation of *Vlv*, resulting in this process being considerably more involved. Thus, the shaded central Region III in Fig. 1 is considerably more complicated than Regions I and II.

While the overall method involves approximating two output waveforms (*V*_*lv*_, *P*_*lv*_) from a single input waveform (*P*_*ao*_), it’s important to note that all three waveforms (*P*_*lv*_, *V*_*lv*_, *P*_*ao*_) have distinct features. Different regions of behaviour are governed by different physiological phenomenon, and these waveforms have been extensively characterised [26]. As such, all three waveforms are heavily interconnected and information rich, making this task more reasonable than it might first appear.

#### Determining *P*_*lv*_ from *P*_*ao*_ (Region I, Fig. 1)

**Fig. 2:**
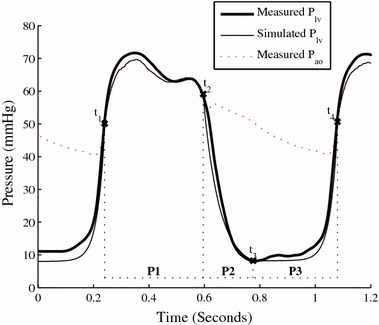
Simulating left ventricular pressure. Note *P*_*ao*_ has been shifted left by *δ* to account for phase lag

The left ventricle is situated directly upstream from the aorta, separated by the aortic valve. This valve is open during systole and closed during diastole. As such, if aortic valve resistance is neglected, *P*_*lv*_ is equivalent to *P*_*ao*_, with a slight phase lag (*δ*) during the majority of systole (Section P. 1, Fig. 2). While aortic valve resistance is non-negligible in conditions such as aortic stenosis [26], valve dysfunction of any type is present in only 3.61% of CVD mortalities in the US [27]. Further, such conditions are typically chronic in nature, relatively easily diagnosed [28] and evolve slowly, while the method is designed to monitor short term changes in an ICU environment. As such, this assumption should not significantly affect the methods’ applicability to the vast majority of the target cohort.

During diastole the aortic valve is closed and little information is available from the aortic waveform about ventricular behaviour. However, the ventricle behaves in a largely passive manner in this region, meaning the TVE curve is typically near zero during diastole [26]. As such, a generic function consisting of two exponentials was used to approximate left ventricular pressure during diastole. In early diastole (Section P. 2, Fig. 2) an exponential decay to a fixed baseline pressure captures ventricular relaxation. In late diastole-early systole (Section P. 3, Fig. 2), an exponential increase captures the beginning of ventricular contraction [26].

While atrial contraction contributes significantly to late diastolic filling, ventricular elastance remains largely passive in late diastole [26]. Thus the TVE curve is typically at its baseline value until the beginning of ventricular contraction in early systole. While atrial function affects the magnitude of the driver function, which is normalised in this work, and may affect its shape, this effect is indirect as the driver function represents the impact of contraction in driving pulsatile blood from the heart to the arterial system. Further, with the increasing unpopularity of pulmonary artery catheters [29] none of the typically available instrumentation in an ICU provides a clear picture of atrial behaviour. As such, while the exponential in section P. 3 is broadly intended to capture ventricular filling, no specific atrial behaviour component is integrated into this model.

Using Fig. 2, the left ventricular pressure for the *n*_*th*_ heartbeat is thus defined using *P*_*ao*_:

2a $$t_{1} = t\left( {\frac{{dP_{ao} }}{dt}_{max} } \right)_{n}$$

2b $$t_{2} = t\left( {\frac{{dP_{ao} }}{dt}_{min } } \right)_{n}$$

2c $$t_{4} = t\left( {\frac{{dP_{ao} }}{dt}_{max } } \right)_{n + 1}$$

2d $$t_{3} = 0.62t_{2} + 0.38t_{4}$$

3 $$P_{lv} (t) = \left\{ {\begin{array}{*{20}l} {P_{ao} \left( {t_{1} + \delta < t < t_{2} + \delta } \right)} \hfill & {t_{1} < t < t_{2} } \hfill \\ {6 + \left( {P_{ao} \left( {t_{2} } \right) - 6} \right)e^{{ - 17.5\left( {t - t_{2} } \right)}} } \hfill & {t_{2} < t < t_{3} } \hfill \\ {P_{lv} \left( {t_{3} } \right) + \left( {P_{ao} \left( {t_{4} } \right) - P_{lv} \left( {t_{3} } \right)} \right)e^{{37.5\left( {t - t_{4} } \right)}} } \hfill & {t_{3} < t < t_{4} } \hfill \\ \end{array} } \right\}$$

where: $$\delta = 0.008{\text{s}}$$

#### Determining *V*_*d*_ from baseline *V*_*es*_ (Region II, Fig. 1)

A recent method has been developed to approximate *V*_*d*_ from ventricular volume measurements [30]. This approach relies on linear regression of the Frank-Starling curve (*SV*–*V*_*ed*_) and its end-systolic equivalent (*SV*–*V*_*es*_) to the point where *SV* = 0 and ‘*the ventricle cannot develop any systolic pressure*’, the definition of *V*_*d*_ [23]. This work also showed *V*_*d*_ for a baseline, healthy pig be an approximately fixed percentage of *V*_*es*_ [30]. Defining *V*_*d*_ as a percentage of baseline *V*_*es*_ allows approximation of baseline *V*_*d*_ during the initial echocardiographic reading where measured *V*_*es*_ is available. While *V*_*d*_ has been shown to change with condition, there is no practical means of capturing this change short of additional echocardiography measurements. Thus, while intermittent measures are feasible, in this study *V*_*d*_ is fixed at a baseline value.

4 $$V_{d} = 0.48 \times V_{es}$$

#### Determining *V*_*lv*_*from P*_*ao*_*, HR and V*_*d*_ (Region III, Fig. 1)

**Fig. 3:**
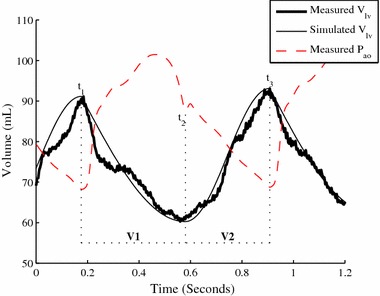
Simulating left ventricular volume

Unlike pressure, little volume or flow information is readily available from the typical, clinically available instrumentation. Simulating *V*_*lv*_ is thus more challenging. The shape of the ventricular volume waveform was approximated by a piecewise sine wave consisting of two sections: systole (Section V. 1, Fig. 3) and diastole (Section V. 2, Fig. 3), with a 90° phase shift at the beginning of systole. The underlying physiological behaviour might be better represented by a series of exponentials [26]. However, using sine waves achieves a similar result with considerably fewer variables involved.

Thus, six points per heartbeat (*t*_1_, *t*_2_, *t*_3_ and (*V*_*ed*_)_*n*_, (*V*_*es*_)_*n*_, (*V*_*ed*_)_*n*+*1*_) are required to define the ventricular volume waveform. The timing associated with systole start (*t*_1_), systole end (*t*_2_), and diastole end (*t*_3_) are readily determined from the aortic pressure waveform (Fig. 3):

5a $$t_{1} = t\left( {P_{ao_{min}} } \right)_{n}$$

5b $$t_{2} = t\left( {P_{DN} } \right)_{n}$$

$$t_{3} = t\left( {P_{ao_{min}} } \right)_{n + 1}$$

Using existing work [31] *SV* can be approximated beat-to-beat using the aortic waveform. Thus, only one of *V*_*es*_ or *V*_*ed*_ is required, as *SV* can be used to convert between the two. The ESPVR allows for determination of *V*_*es*_, and is defined [23]:

6 $$P_{es} = E_{es} \times \left( {V_{es} - V_{0} } \right)$$

where *E*_*es*_ is the end-systolic elastance and *V*_0_ is the ventricular volume at zero pressure. Equation 6 can be rewritten:

7 $$P_{DN} = E_{es} \times \left( {V_{es} - V_{d} } \right)$$

where this change is justified by:

- The pressures in the ventricle and aorta are roughly equivalent until the aortic valve closes, thus *P*_*DN*_ is close to *P*_*es*_
- *V*_*d*_ and *V*_0_ have similar, but distinct, physiological significance and values. The two are often used interchangeably [17, 18, 23]

Finally, it is necessary to account for *E*_*es*_, which changes in response to a number of factors including contractility [6], and loading conditions [32, 33]. Thus, Eq. 7 is modified:

8 $$P_{DN} = \left( {E_{c} \times HR^{3} } \right) \times \left( {V_{es} - V_{d} } \right)$$

here *E*_*es*_ is defined as a function of *HR* and a coefficient (*E*_*C*_), with a cubic selected as it provides the best compromise between simplicity and effective tracking for the data set presented here. In particular, the cardiovascular system responds to most changes in conditions in a number of ways, including changes in heart rate and elastance. As heart rate is easily measured, it provides an easy to obtain, if incomplete, indication of cardiovascular system response, which can be used to inform an approximated elastance [34]. Further supporting evidence is provided in the validation and discussion of results.

During the echocardiography calibration, measurements for *P*_*DN*_, *HR* and *V*_*es*_ are available [35]. Thus, using Eq. 8, a constant value for *E*_*C*_ can be defined, allowing approximation of *E*_*es*_ and thus determination of *V*_*es*_ on a beat-by-beat basis. The beat-to-beat ventricular volume can thus be determined:

9 $$V_{lv} (t) = \left\{ {\begin{array}{*{20}l} {\left( {V_{ed} } \right)_{n} + \left( {\left( {V_{es} } \right)_{n} - \left( {V_{ed} } \right)_{n} } \right)\sin \left( {\frac{{\pi \left( {t - t_{1} } \right)}}{{2\left( {t_{2} - t_{1} } \right)}}} \right)} \hfill & {t_{1} < t < t_{2} } \hfill \\ {\left( {V_{es} } \right)_{n} - \left( {\left( {V_{ed} } \right)_{n + 1} - \left( {V_{es} } \right)_{n} } \right)\left( {\frac{1}{2}\cos \left( {\frac{{\pi \left( {t - t_{2} } \right)}}{{\left( {t_{3} - t_{2} } \right)}}} \right) - \frac{1}{2}} \right)} \hfill & {t_{2} < t < t_{3} } \hfill \\ \end{array} } \right\}$$

where:

10 $$V_{es} = \frac{{P_{DN} }}{{\left( {E_{c} \times HR^{3} } \right)}} + V_{d}$$

11 $$V_{ed} = V_{es} + SV$$

#### Summary of proposed method

The overall derivation of the TVE curve can be summarised:

#### Initially or intermittently

1. Calculate *V*_*d*_ using Eq. 4 and baseline *V*_*es*_ (Region I, Fig. 1)
2. Calculate *E*_*C*_ using Eq. 8, *P*_*DN*_, *HR* and baseline *V*_*es*_ (Region III, Fig. 1)

#### Every heartbeat

1. Simulate *P*_*lv*_ using Eq. 2, Eq. 3 and *P*_*ao*_ (Region II, Fig. 1)
2. Determine *V*_*es*_ using Eq. 10, *P*_*DN*_, *HR* and *E*_*C*_ (Region III, Fig. 1)
3. Determine *SV* using [31] and *P*_*ao*_ (Region III, Fig. 1)
4. Determine *V*_*ed*_ using Eq. 11, *V*_*es*_ and *SV* (Region III, Fig. 1)
5. Simulate *V*_*lv*_ using Eq. 5, Eq. 9, *P*_*ao*_, *V*_*es*_ and *V*_*ed*_ (Region III, Fig. 1)
6. Calculate and normalise the TVE curve *e*(*t*) using Eq. 1

### Analysis and validation

The proposed method was validated on experimentally gathered data. A range of input (*P*_*ao*_) and output (*V*_*lv*_, *P*_*lv*_) waveforms were continuously measured via catheter. This data allowed validation of individual model assumptions through comparison with directly measured output waveforms, as well as validation of the overall method through comparisons between the TVE curve as calculated using simulated and directly measured *V*_*lv*_ and *P*_*lv*_ wave forms.

The data set encompasses 46,318 heartbeats across 5 Piétrain pigs. A diverse clinical protocol provides the ability to assess intra- and inter-subject variability across a large amount of invasive and non-invasive measurements. Together they enable rigorous assessment and validation of the method.

#### Experimental procedure

**Fig. 4:**
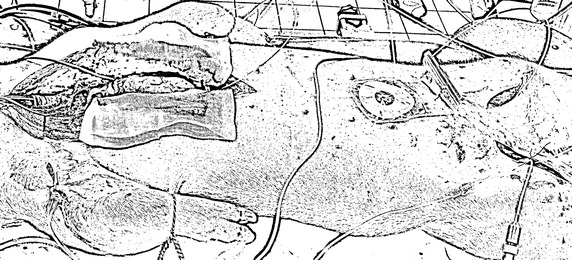
The fully instrumented Piétrain Pig. The catheters for measuring *P*_*ao*_, *P*_*lv*_ and *V*_*lv*_ are positioned to the left of the image

The experimental protocol was approved by the Ethics Commission for the Use of Animals at the University of Liège, Belgium. Five male, pure Piétrain pigs weighing between 18.5 and 29 kg were sedated, anaesthetised and mechanically ventilated (GE Engstrom CareStation) with a baseline positive end-expiratory pressure (PEEP) of 5 cmH_2_O (Fig. 4, Additional file 1: Table S1). Proximal aortic pressure was continually sampled using a pressure catheter (Transonic, NY, USA) with a sampling rate of 250 Hz. To provide direct measurements of *P*_*lv*_ and *V*_*lv*_ for validation, the heart was accessed via a median sternotomy, and an admittance pressure–volume catheter (Transonic, NY, USA) with a sampling rate of 250 Hz inserted into the left ventricle via an apical stab [36, 37].

To demonstrate a diverse range of cardiac states, several procedures were performed:

- A single infusion of endotoxin (lipopolysaccharide from *E. Coli*, 0.5 mg/kg injected over 30 min) to induce septic shock. Septic shock drives a change in afterload conditions and is associated with a large variety of effects including an inflammatory response and capillary leakage that may lead to hypovolemia, decreased cardiac output, decreased ejection fraction and cardiac failure [38].
- Several PEEP driven recruitment manoeuvres (RMs), both pre- and post-endotoxin infusion. RMs drive a change in preload conditions and are typically associated with a decrease in mean blood pressure and cardiac output [39].
- One to four infusions of 500 mL saline solution over 30 min, pre- and post-endotoxin infusion, simulating fluid resuscitation therapy, a key component of hemodynamic resuscitation in patients with severe sepsis, which itself results in a change in circulatory volume [40].

#### Validation of significant model assumptions

Two major assumptions made in deriving this model are:

1. That *V*_*d*_ can be expressed as a function of baseline *V*_*es*_, as in Eq. 4
2. That *E*_*es*_ can be expressed as a function of *HR*, as in Eq. 8

Direct evaluation of the tracking of *V*_*es*_ using different forms of ESPVR allows validation of both of these assumptions. In particular, 3 different methods of tracking *V*_*es*_ were compared:

1. **Fixed** ***E***_***es***_ **and neglected** ***V***_**0**_: The standard ESPVR (Eq. 6) with *V*_0_ = 0 (a commonly used assumption [17, 18, 23])
2. **Fixed** ***E***_***es***_ **and fixed** ***V***_**0**_: The standard ESPVR (Eq. 7) with *V*_0_ = *V*_*d*_ (allowing assessment of the validity of Eq. 4)
3. **Dynamic** ***E***_***es***_ **and fixed** ***V***_**0**_: The ESPVR as used in the proposed method (Eq. 8) with *V*_0_ = *V*_*d*_, and *E*_*es*_ as a function of *HR* (allowing assessment of the validity of Eq. 8)

#### Validation of overall model

**Fig. 5:**
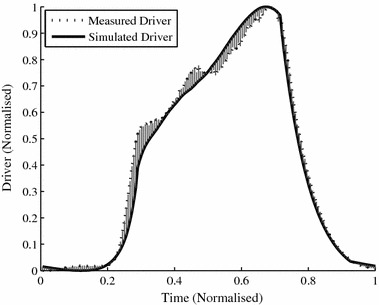
Example TVE curve error. This simulated driver has an error of 7.8% and bias of −3.4%

The overall method presented here is designed to simulate the TVE curve beat-by-beat, without requiring invasive instrumentation of the heart or real-time image-based monitoring, neither of which is clinically or ethically feasible in care. As such, validation of the method relies on comparison of the simulated TVE curve to the invasively measured, ‘true’ TVE curve, which is calculated using the catheter measured *V*_*lv*_ and *P*_*lv*_ waveforms for a single beat. This comparison is achieved by calculating the absolute and signed ‘error area’ between the measured and simulated TVE curve, according to Eqs. 12 and 13:

12 $$\varepsilon_{abs} = \frac{{\mathop \smallint \nolimits_{t = 0}^{1} \left| {e_{sim} (t) - e_{meas} (t)} \right|}}{{\mathop \smallint \nolimits_{t = 0}^{1} \left( {e_{meas} (t)} \right)}}$$

13 $$\varepsilon_{\text{sgn}} = \frac{{\mathop \smallint \nolimits_{t = 0}^{1} \left( {e_{sim} (t) - e_{meas} (t)} \right)}}{{\mathop \smallint \nolimits_{t = 0}^{1} \left( {e_{meas} (t)} \right)}}$$

where *ε*_*sim*_ and *ε*_*meas*_ are the simulated and measured TVE curves respectively, *t* is normalised time set to 1 for every heart beat to enable comparison over different beats, and *ε*_*abs*_ and *ε*_*sgn*_ and denote the absolute and signed errors respectively. An example TVE curve with an absolute error of 7.8% and bias of −3.4% is shown in Fig. 5, where shading denotes the error area.

## Results

### Validation of significant model assumptions

Table 1 shows the percentage error associated with the 3 methods specified to track *V*_*es*_. Moving from neglected *V*_0_ (Method 1) to fixed *V*_0_ (Method 2), via Eq. 4, shows a modest reduction in median and 25th percentile errors (15.9–14.5% and 3.1–1.3%, respectively), but a highly significant reduction in 75th percentile error (43.2–25.5%). Introduction of dynamic *E*_*es*_ (Method 3) as opposed to fixed *E*_*es*_ (Method 2), via Eq. 8, shows a very significant reduction in median and 75th percentile errors (14.5–4.3% and 25.5–15.0%, respectively). These results support the validity and usefulness of Eqs. 4 and 8, their associated assumptions in the tracking of *V*_*es*_, and thus the use of Method 3 in the overall approximation of the TVE curve.

**Table 1:** Absolute percentage error associated with different methods of tracking V_es_

Pig	1. Fixed *E*_*es*_, neglected *V*_0_			2. Fixed *E*_*es*_, fixed *V*_0_			3. Dynamic *E*_*es*_, fixed *V*_0_		
	25th Perc. (%)	Med (%)	75th Perc. (%)	25th Perc. (%)	Med (%)	75th Perc. (%)	25th Perc. (%)	Med (%)	75th Perc. (%)
Pig 1	0.7	4.6	23.9	0.6	8.7	14.7	0.2	1.8	9.0
Pig 2	2.1	19.5	39.1	2.1	23.4	34.0	1.8	6.5	16.5
Pig 3	4.2	12.2	45.0	0.2	5.6	16.6	0.5	4.5	12.7
Pig 4	7.3	30.7	42.7	2.9	23.3	29.3	0.4	4.7	13.6
Pig 5	1.1	12.4	65.4	0.9	11.7	32.7	0.4	4.2	23.4
Mean	3.1	15.9	43.2	1.3	14.5	25.5	0.7	4.3	15.0

Note the values quoted from the final row of Table 1 are an average of the 25th percentile, median and 75th percentile error for the pigs rather than an overall 25th percentile, median and 75th percentile error. The decision was made to represent population values in this way as the overall median is less representative than the average median due a differing number of heartbeats recorded for different pigs.

**Fig. 6:**
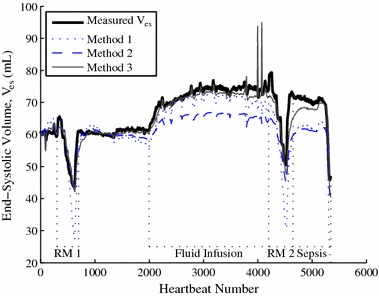
Tracking of *V*_*es*_. Comparison between the three methods in 2.2.2 for Pig 1

The comparative behaviour of these 3 methods is well illustrated in Fig. 6, which shows the tracking of *V*_*es*_ for Pig 1. Method 1 agrees reasonably well with measured *V*_*es*_ during normal behaviour, but diverges significantly from measured *V*_*es*_ during recruitment manoeuvres and the onset of severe sepsis, where there are large vertical changes in *V*_*es*_. Method 2 accurately captures these large vertical changes in *V*_*es*_, reducing the large 75th percentile error in Method 1. Method 3 retains the tracking of large vertical changes in Method 2, and tracks normal behaviour more effectively by accounting for changes in *E*_*es*_, significantly reducing median error.

### Validation of overall model

The overall purpose of this model is to track the shape of the TVE curve, and how this shape changes when circulatory behaviour changes. Table 2 shows the area under the curve errors, *ϵ*_*abs*_ and *ϵ*_*sgn*_, for all 5 pigs. The median error is relatively small at 11.4%, suggesting the method is effective, and the interquartile range relatively narrow at 9.2–14.7%, suggesting the method is consistent. The bias is also small at −2.5%, with an interquartile range of −6.1 to 0.8%, suggesting only a slight method bias.

**Table 2:** TVE curve percentage area under the curve errors (*ε*_*abs*_ and *ε*_*sgn*_) associated with proposed method (identifying *V*_*es*_ and *V*_*ed*_)

Pig	Absolute percentage error, *ε*_*abs*_			Signed percentage error, *ε*_*sgn*_		
	25th Perc.	Med	75th Perc.	25th Perc.	Med	75th Perc.
Pig 1	9.0	11.6	14.4	−7.0	−4.9	−2.6
Pig 2	7.8	9.4	11.6	−1.4	2.3	6.6
Pig 3	9.4	11.4	15.6	−12.6	−7.8	−4.5
Pig 4	9.5	11.1	13.0	−9.3	−7.5	−5.7
Pig 5	10.5	13.4	19.0	−0.3	5.2	10.1
Mean	9.2	11.4	14.7	−6.1	−2.5	0.8

Table 3 shows, as a point of comparison, the area under the curve errors associated with the method if the directly measured values for *V*_*es*_ and *V*_*ed*_ are used. Using these measured values removes the majority of Region III, Fig. 1 (Eqs. 4 and 6–11) from the overall method, where a lot of relatively significant assumptions are made. The errors in Table 3 are very comparable to those in Table 2, with an overall modest reduction in average median error (11.4–10.2%) and bias (−2.5 to −2.2%), as expected when measuring *V*_*es*_ and *V*_*ed*_ directly and invasively rather than estimating them. Hence, the assumptions made had very little impact on error when removed.

**Table 3:** TVE curve percentage area under the curve error (*ε*_*abs*_ and *ε*_*sgn*_) associated with using measured *V*_*es*_ and *V*_*ed*_ values

Pig	Absolute percentage error, *ε*_*abs*_			Signed percentage error, *ε*_*sgn*_		
	25th Perc.	Med	75th Perc.	25th Perc.	Med	75th Perc.
Pig 1	9.0	11.5	13.3	−8.1	−5.6	−2.7
Pig 2	7.3	8.5	10.1	−3.7	−0.7	2.2
Pig 3	7.1	8.1	9.1	−4.7	−2.7	−0.6
Pig 4	9.5	11.4	13.5	−9.9	−7.8	−5.6
Pig 5	9.8	11.3	13.5	2.8	6.0	9.0
Mean	8.5	10.2	11.9	−4.7	−2.2	0.5

Figure 6 shows a variety of measured and modelled TVE curve shapes for each of the 5 pigs, capturing both intra- and inter-subject variability. For each figure, Panel I shows a baseline waveform, Panel II a waveform during a RM and Panel III a waveform after endotoxin infusion has occurred and sepsis develops. The range of shapes and inter-pig variability indicated that assuming a generic population or cohort TVE curve is not particularly valid, and is further supported by the range of intra-pig variability evidenced. There are a variety of shoulder heights, relative gradient and maximum/minimum gradient timings that result in TVE curves with distinct shapes, variations which are well captured by the method. These TVE curves were also selected to demonstrate a range of error values similar to the interquartile error range for that pig (Table 2). Thus, these drivers are reasonably representative of the overall ability of the method to capture TVE curve shapes, despite different overall shapes for each pig and changes in shape as condition changes.

## Discussion

### Validation of significant model assumptions (Table 1)

A direct evaluation of the effectiveness of three different ESPVR equations set out in 2.2.2 in tracking *V*_*es*_ allowed validation of the assumptions that:

- That *V*_*d*_ can be derived from *V*_*es*_ as in Eq. 4 (implemented in Method 2)
- That *E*_*es*_ can be expressed as a function of *HR* as in Eq. 8 (implemented in Method 3)

Method 1 serves as a control and uses a simplified ESPVR, assuming *V*_0_ = 0 and *E*_*es*_ is constant. This method tracks *V*_*es*_ reasonably well, yielding an overall median error of 15.9% across all 5 pigs compared to *V*_*es*_ as directly measured. The assumption that *V*_0_ can be neglected is often used due to a combination of *V*_0_ being difficult to measure directly, as it requires a significant artificial reduction in ventricular pressure [41], and *V*_0_ typically being relatively small [42]. This assumption is largely supported by these results, as a 15.9% median error seems acceptable when weighed against the type of highly invasive and involved protocol traditionally required to determine *V*_0_.

However, as shown in Fig. 6, this 15.5% median error fails to capture the extremely large inaccuracies associated with using Method 1 to track sudden changes in *V*_*es*_ due, for example, to recruitment manoeuvres or the onset of severe sepsis. This failure to track sudden changes leads to a significantly larger 75th percentile error of 43.2% for Method 1. This high error when sudden changes occur is of concern in an ICU or cardiac surgery clinical scenario, where sudden changes and accurate, rapid determination of patient responses to these sudden changes is extremely important [26].

Method 2 introduces the assumption that *V*_*d*_ can be derived from baseline *V*_*es*_, as in Eq. 4, and that *V*_*d*_ can be used as a surrogate for *V*_0_. There is a minor, but important physiological distinction between the two values: *V*_0_ is the ventricular volume at 0 pressure, while *V*_*d*_ is the volume at which the ventricle cannot develop any systolic pressure [23]. However, the purpose of both terms is to account for the subject-specific inactive volume within the ventricle, and the two values have been shown to be similar for a given subject [23].

Method 2 results in a notable decrease in 75th percentile error compared to Method 1 (43.2–25.5%), and is able to track sudden changes in *V*_*es*_ significantly more effectively, as shown in Fig. 6. However, Method 2 yields only a modest reduction in median error compared to Method 1 (15.9–14.5%), as both methods fail to capture the dynamic nature of *E*_*es*_. Thus, large errors are reduced, but overall accuracy is not greatly improved. Regardless, these results provide support for the validity of Eq. 4, and the use of *V*_*d*_ as a surrogate for *V*_0_ here. The fact that these reductions in error are sustained over significant changes in cardiac output and ejection fraction as sepsis develops suggests that the absolute value of *V*_*d*_ does not change significantly enough under such conditions to detract from method accuracy.

Method 3 further assumes that *E*_*es*_ can be expressed as a function of *HR.* This assumption is unusual, but is supported by the results. The full method sees a further, significant reduction in 75th percentile error compared to Methods 1 and 2 (43.2% and 25.5–15.0%, Table 1), and, most importantly, a very large reduction in median error compared to both Methods 1 and 2 (15.9% and 14.5–4.3%, Table 1). This result suggests general tracking of trends in *V*_*es*_ is being significantly improved, also supporting the validity of expressing *E*_*es*_ as a function of *HR.* This behaviour can also be observed in Fig. 6. This result, combined with the minimal addition in method complexity required to include *HR*, which is very easy to measure, provides a strong case for the use of Method 3.

However, it is still important to note that the relationship between *E*_*es*_ and *HR* expressed in Eq. 8 is a significant simplification of actual cardiac behaviour. The cardiac system uses a large variety of responses to maintain cardiac output. The cubic used to approximate *E*_*es*_ changes as a function of *HR* attempts to mathematically approximate the sympathetic nature of some of these responses, but, inevitably, the relationship between *HR* and *E*_*es*_ varies between subjects (accounted for by calibration), and as time and condition changes. While the cubic approximation of this relationship has been demonstrated to remain effective across the full progression of septic shock in the data presented here, further validation of this relationship, and especially the use of a cubic, across other conditions is desired.

### Validation of overall model

**Fig. 7:**
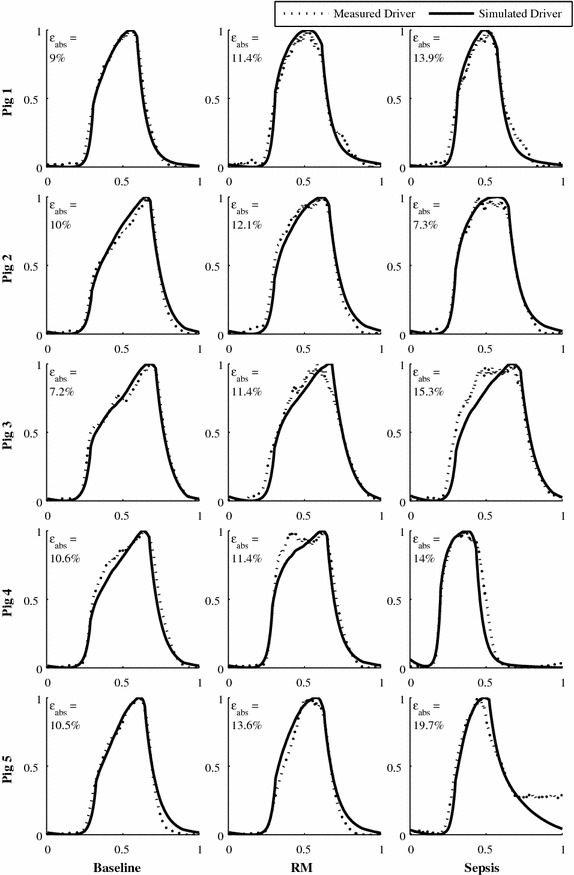
Example TVE curves for each pig. A range of error values and cardiac states are shown

Simulating the TVE curve using the proposed method resulted in a relatively low median absolute error area, ranging from 9.4 to 13.4% (Table 2) across all pigs. This narrow range of median absolute errors implies the method is able to consistently and effectively capture inter-subject variations in TVE curve behaviour, suggesting it is generalizable to other subjects. This inter-subject variability is demonstrated well by various baseline drivers shown in Fig. 7. All 5 pigs demonstrate relatively distinct baseline TVE curve shapes, showing a considerable level of inter-subject variability impossible to capture with a generic cohort or population based TVE curve that relies on a basic assumed shape [17, 18].

The simulated TVE curve also had a relatively narrow average interquartile error range of 9.2–14.7% (Table 2), suggesting the method is able to relatively consistently and effectively capture inter-subject variations in TVE curve behaviour. This intra-subject variability, and inter-quartile error range, is illustrated in Fig. 7. Accurate capture of relative changes and trends in patient behaviour is extremely important, as these changes are critical in assessing whether a patient is recovering, responding to treatment, or is in need of a change in treatment. Most pigs demonstrated notable changes and intra-pig variability in driver shape during recruitment manoeuvres and as sepsis developed. A couple of the more unusual driver shapes were not fully captured, for example Panel 2 of Pig 4 in Fig. 7, where the driver measured driver displayed two peaks and the simulated driver only one.

The simulated TVE curve demonstrated consistently low signed area error, at −2.5% (IQR −6.1 to 0.8%), shown in Table 2. This result shows the method only slightly underestimating the TVE curve, supported further by 2 pigs having a slight positive bias and the other 3 a slight negative bias. This outcome further supports the ability of the TVE curve to accurately capture both intra- and inter-subject variability over time and condition.

In assessing the impact of assumptions on the TVE curve, a comparison of Table 2, using approximated *V*_*es*_ and *V*_*ed*_, and Table 3, using measured *V*_*es*_ and *V*_*ed*_, show very similar error values. For example, overall median error fell from 11.4 to 10.2% and overall bias from −2.5 to −2.2%, only a modest reduction in error. This implies that the body of assumptions and equations in Region III, Fig. 1 concerned with the approximating the *V*_*es*_ and *V*_*ed*_ for simulation of *V*_*lv*_ do not result in a large increase in error compared to using measured *V*_*es*_ and *V*_*ed*_. The assumptions made in areas of the method not involving simulating *V*_*es*_ and *V*_*ed*_ are relatively minimal, mostly involving using *P*_*ao*_ to determine waveform timing. As such, it would seem that much of the error associated with this method is the result of the necessity of assuming equations for parts the two waveforms being reconstructed (*P*_*lv*_ and *V*_*lv*_), as shown in Figs. 2 and 3. Thus, the assumptions employed in Region III, Fig. 1 appear to function as intended, and further reduction in error would probably require increased method complexity or an increase in the clinically available data.

An important point to consider is the fact that the TVE curve is consistently normalised to a duration and amplitude of 1.0, as it is designed specifically as an indicator of how the heart is behaving relatively over the course of a beat, to be coupled with a lumped metric (*E*_*es*_) indicating the overall strength of that heartbeat [6, 17, 18]. This normalisation does mean that some of the errors associated with the various assumptions and approximations made throughout the method are negated, and that indicators of absolute cardiac work and its changes, for example, are not able to be directly extracted from the TVE curve created. Fortunately, the driver does not exist alone, the intent is that the shape of the TVE curve, indicative of transient, relative cardiac behaviour, be used alongside other existing metrics, such as Cardiac Output or Stroke Volume [31, 43], indicative of lumped, absolute cardiac behaviour, to provide further diagnostic information.

### Limitations

There are study limitations that should be considered. First, all data presented is derived from a single protocol involving a single, but complex and varied [38], condition (sepsis). This data set encompasses several pigs, a full progression from healthy, baseline behaviour to cardiac failure and clinically standard ventilation and fluid interventions. Nevertheless, there is a much larger range of possible cardiac conditions, and further validation over several of these would be beneficial. For example, the method would benefit from validation on contractility altering drugs such as dobutamine [44], which may alter the behaviour of the elastance term in Eq. 8. However, the method already detects changes in haemodynamics, including those due to circulatory or cardiac muscle changes during sepsis in this study. Thus, the ability to detect changes due to inotropes should be similar to what is presented for the range of behaviours already observed in sepsis. Further, given inotrope infusions are determined and performed by a clinician, it would be possible to recalibrate the method directly after such an infusion to adjust to the new inotropic state if it proved necessary. Overall, the underlying physiology and data supporting the development of this method has been discussed in detail, and would be expected to generalise well to a wider range of conditions, as there are no intervention or condition specific assumptions made.

The method also requires validation on human subjects to ensure the methodology as presented here remains physiologically accurate, though the strong similarities between porcine and human physiology and the effectiveness of porcine models are well established [45, 46]. Equally, only an animal model, as used here, allows the direct validation against cardiac measured PV loops, which would not be possible in humans. Thus, only an animal trial allows this important first validation.

The method does require an initial calibration via echocardiography or similar means. Echocardiography equipment is increasingly available in modern ICUs [25]. Further, echocardiography is non-invasive and the calibration period required is relatively short, requiring approximately 10 heartbeats. However, the requirement of such a calibration still prevents the method from being fully implementable without modest additional clinical workload using normal ICU instrumentation.

## Conclusion

The TVE curve is an important, but difficult to clinically measure, expression of internal cardiac dynamics that captures the heart’s ability as a pump and can evolve over time, condition and patients. A novel, minimally invasive method for deriving the TVE curve beat-by-beat, by combining simple physiological assumptions with readily available catheter waveforms to individually simulate the components of the TVE curve, is proposed. This method was assessed across a cohort of 5 Piétrain pigs undergoing a progression from healthy behaviour to cardiac failure due to sepsis. The TVE curve generated by the method was shown to effectively track a directly measured function throughout the experiments, with low overall median absolute (11.4%) and signed (−2.5%) area under the curve errors. There is the potential for this method to provide real time, patient specific information on intra-beat behaviour and inter-beat variation in the heart, at the patient bedside, without requiring additional, invasive instrumentation.

## Additional file

**Additional file 1: Table S1.** Supplementary Subject Specific Experimental Information.

## Abbreviations

CVD: cardiovascular disease and dysfunction
ICU: Intensive Care Unit
ESPVR: end-systolic pressure–volume relation
P–V: pressure–volume
PEEP: positive end-expiratory pressure
RM: recruitment manoeuvre
