# Supplementary material for: Minimally invasive, patient specific, beat-by-beat estimation of left ventricular time varying elastance
Source: Biomed Eng Online. 2017 Apr 13;16:42. doi: 10.1186/s12938-017-0338-7 (PMC5390429; doi:10.1186/s12938-017-0338-7)
Supplement: Supplementary file 1 — Additional file 1: Table S1. Supplementary Subject Specific Experimental Information. [file 12938_2017_338_MOESM1_ESM.docx]

# Appendix A. Additional file 1

**Table S1: Supplemental, Subject Specific Physiological Information**

| **Pig** | **Weight (kg)** | **Baseline Volume (mL)** | **Max Aortic Pressure (mmHg)** | | |
| --- | --- | --- | --- | --- | --- |
|  |  |  | 25^th^ Perc. | Med | 75^th^ Perc. |
| Pig 1 | 27 | 60.6 | 70.6 | 72.9 | 77.6 |
| Pig 2 | 29 | 49.3 | 63.8 | 66.1 | 68.6 |
| Pig 3 | 27 | 61.7 | 68.3 | 81.2 | 97.2 |
| Pig 4 | 25 | 52.6 | 49.2 | 53.6 | 56.1 |
| Pig 5 | 26 | 39.4 | 50.2 | 57.7 | 60.7 |
